# Psychological conditions of caregivers of adult subjects with Prader-Willi syndrome

**DOI:** 10.1186/s13023-024-03385-6

**Published:** 2024-10-22

**Authors:** Anna Guerrini Usubini, Adele Bondesan, Diana Caroli, Francesca Frigerio, Graziano Grugni, Gianluca Castelnuovo, Alessandro Sartorio

**Affiliations:** 1 Istituto Auxologico Italiano, Istituto di Ricovero e Cura a Carattere Scientifico (IRCCS), Experimental Laboratory for Auxo-Endocrinological Research, Piancavallo-Verbania, 28824 Italy; 2 Istituto Auxologico Italiano, Istituto di Ricovero e Cura a Carattere Scientifico (IRCCS), Psychology Research Laboratory, Milan, 20145 Italy; 3 Department of Psychology, Catholic University of Milan, Milan, 20123 Italy

**Keywords:** Prader-Willi syndrome, Caregivers, Psychological distress, Psychological well-being, Coping

## Abstract

**Background:**

Prader-Willi syndrome (PWS) is a rare genetic neurodevelopmental disorder. Individuals with PWS face a range of cognitive, behavioral, and emotional challenges that require comprehensive and lifelong care, posing significant demands on their caregivers. The study is not only aimed to assess the psychological conditions of caregivers of adult subjects with PWS focusing on psychological distress and coping, but also to shed light on a crucial yet often overlooked aspect of healthcare. This study aims to compare the psychological well-being of individuals with PWS and their caregivers, providing valuable insights that can potentially improve the quality of care for these individuals. The sample recruited at the Division of Auxology, IRCCS Istituto Auxologico Italiano, was composed of 30 adult subjects with PWS (11 men and 19 women; mean age ± SD: 36.4 ± 10.31 years; mean Body Mass Index (BMI): 35.7 ± 8.92: kg/m2) and their caregivers (10 men and 20 women). To assess the psychological condition of caregivers, the Italian-validated versions of the Depression Anxiety and Stress Scale (DASS-21) and the Coping Orientation to the Problems Experiences (COPE) were used, while to assess the psychological well-being of individuals with PWS and their caregivers, the Italian validated version of the Psychological General Well-Being Index (PGWBI) was used.

**Results:**

Depression (*p* < 0.001), Stress (*p* = 0.050), and Total score (*p* = 0.009) of DASS 21 were higher in the caregivers of subjects with PWS than in the general population. PGWBI scores of caregivers were significantly lower than in individuals with PWS in Positive Well-being (*p* < 0.001), General Health (*p* = 0.006), Vitality (*p* = 0.004), and the total score (*p* = 0.006). The depression subscale of PGWBI was higher in caregivers than in subjects with PWS. Correlations between the subscales of COPE and the total score of PGWBI in caregivers revealed that the Avoidance subscale of COPE had a negative significant correlation with the total score of PGWBI (*p* = 0.003).

**Conclusions:**

Our results highlighted several critical insights into the profound emotional and psychological challenges faced by the caregivers of individuals with PWS.

## Background

Prader-Willi syndrome (PWS) is a rare genetic neurodevelopmental disorder caused by the lack of expression of paternally inherited imprinted genes on chromosome 15q11–q13 [1, 2]. It is primarily characterized by hypotonia and poor feeding in early infancy, followed by excessive eating and the gradual development of morbid obesity from early childhood if uncontrolled). Multiple endocrine abnormalities, such as hypogonadism and short stature, intellectual disabilities, and other critical psychological features also characterize the PWS phenotype [3]. Individuals with PWS face a range of cognitive, behavioral, and emotional challenges. Cognitive impairments, including mild to moderate intellectual disabilities, impact their learning abilities and everyday functioning. Behavioral issues, such as controlling and compulsive behaviors, negatively affected their interactions [4]. Mood disorders like anxiety and depression are prevalent among this population, often exacerbated by social isolation and the constant need for routine and structure. Some adults may also experience psychotic symptoms, adding another layer of complexity to their psychological profile [5].

Individuals with PWS require comprehensive and lifelong care, posing significant demands on their caregivers. Caregivers, often family members, face several challenges due to the complex and multifaceted nature of the syndrome. They are required to deal with many daily life issues, such as managing hyperphagia and obesity, addressing behavioral and psychiatric problems, and providing support for intellectual disabilities of individuals with PWS [6].

The burden on caregivers is substantial, encompassing physical, emotional, and financial dimensions [7]. Many studies have assessed the burden of caregiving by examining caregiver psychological distress and coping, reporting higher levels of stress and mood disturbances, and difficulties in coping strategies in caregivers dealing with their child’s problems [8](The cumulative effect of these burdens significantly impacts the quality of life for caregivers, as well as their health and well-being. This, in turn, can affect the quality of care provided to the individual with PWS, creating a cycle of increasing stress and burden [9]. In this regard, coping style is a particularly relevant factor associated with the management of PWS.

Literature has traditionally conceptualized coping as how people respond and act when experiencing stress [10]. Coping strategies are individuals’ cognitive and behavioral efforts to interpret and overcome problems and challenges [11, 12]. Some coping strategies may be related to solving problems, changing the situation, and changing the source of stress (problem-focused coping strategies). In contrast, others are related to emotional responses to stress (emotional-focused coping strategies) [13]. In particular, avoidance coping involves cognitive and behavioral efforts oriented toward denying, minimizing, or otherwise avoiding dealing directly with stressful demands [14]. Coping plays a vital role in modulating the physical and psychological burden in caregiving [15].

As mentioned by Shivers and colleagues [16], caring for individuals with PWS requires the adoption of active coping strategies by the caregivers to deal with their needs. On the contrary, avoidant coping strategies may be particularly detrimental for caregivers of individuals with PWS, also influencing their quality of life and satisfaction.

Although it is clear that caregivers play a critical role in the well-being of individuals with PWS, there is a relative lack of research focused on their experiences and needs. In addition, most of the research focused their attention on caregivers of children with PWS [6, 17, 18], while less is known about the experience of taking care of adults with PWS [19].

Nevertheless, understanding the potential negative impact of caregiving for individuals with PWS is essential because the burden and lack of well-being that caregivers experience can reach clinical levels for the caregivers and directly impact the quality of care and the outcome for the individuals with PWS [20].

The current study aims to assess the psychological conditions of caregivers of adult individuals with PWS focusing on psychological distress and coping and comparing the levels of psychological well-being in caregivers and their relatives with PWS. In addition, we explored possible associations between coping strategies and well-being in caregivers, addressing the hypothesis that negative coping strategies could have a negative association with psychological well-being.

## Methods

### Participants and procedures

The sample was composed of 30 adult individuals with PWS (11 men and 19 women; mean age ± SD: 36.4 ± 10.31 years; mean Body Mass Index (BMI): 35.7 ± 8.92: kg/m^2^) and their caregivers (11 men: mean age ± SD: 65.4 ± 9.04; BMI: 25.8 ± 3.76 kg/m^2^; 19 women: mean age ± SD: 65.1 ± 9.40; BMI: 25.4 ± 4.85 kg/m^2^). The mean BMI of the caregivers was significantly lower than the BMI of the subjects with PWS (*p* < 0.001) and no correlations were found between the BMI of the subjects with PWS and those of the caregivers (*p* = 0.154). Participants with PWS were consecutively recruited at the Division of Auxology, IRCCS Istituto Auxologico Italiano, an Italian third-level clinical center for subjects with PWS. Only participants of both sexes, older than 18 years of age, with a genetically confirmed diagnosis of PWS who achieved a score > 24 on the Mini-Mental State Examination (MMSE) [21], which warranted an intellectual level allowing appropriate compliance, were included. Participants were excluded if they had any physical or intellectual disability that could compromise their participation in the study.

All but two caregivers/respondents were the parents of the subjects with PWS (9 fathers and 19 mothers). In one case, the caregiver/respondent was the aunt, in another case the caregiver/respondent was the brother.

Eleven subjects with PWS lived only with their parents; five lived with their parents and one or more brothers/sisters. One subject with PWS lived with their parents and the grandmother; seven subjects lived with only one of the parents; and three lived with one of the parents and one or more brother/sister. In a few cases, the subject with PWS lived with their uncles; one lived alone; and one lived in a residential structure.

All subjects with PWS showed the typical clinical phenotype [1] Twenty-four subjects had an interstitial deletion of the proximal long arm of chromosome 15 (del15q11-q13), while six subjects had uniparental maternal disomy for chromosome 15. Nine subjects with PWS were treated with recombinant growth hormone (rec-GH) therapy; four individuals were undergoing thyroxine therapy; and ten subjects were treated with sex steroid replacement therapy. Three subjects received thyroxine and sex steroids; one subject was treated with rec-GH and thyroxine; one with rec-GH, thyroxine, and sex steroids; one individual was treated with rec-GH and sex steroids. Thirteen subjects with PWS received psychiatric therapy for mood or behaviors.

The mean MSSE score of subjects was 26.6 ± 1.92, with all subjects having a score > 24 [21].

The administration of MSSE was done under the supervision of a member of our research team, who verified the patient’s understanding of the questions.

Once informed about the study and after obtaining written informed consent to participate from all subjects and their caregivers, the subjects were screened for eligibility criteria upon their admission to the Institute. Once enrolled, all the participants were asked to complete self-report questionnaires to assess our variables of interest. The study was approved by the Ethical Committee of Istituto Auxologico Italiano, IRCCS, Milan, Italy (approval number: CE: 2023_03_21_02; acronym: PROPSICOPWS). The research was carried out according to the Declaration of Helsinki and its advancements.

### Measures

To assess caregivers’ psychological condition, the Italian-validated versions of the Depression Anxiety and Stress Scale (DASS-21) [22] and the Coping Orientation to the Problems Experiences (COPE) [23] were used. The Italian-validated version of the Psychological General Well-Being Index [24] was used to assess the psychological well-being of individuals with PWS and their caregivers.

The DASS-21 [22] is a self-report questionnaire comprising 21 items rated on a 4-point Likert scale ranging from 0 to 3. It assesses three dimensions: Anxiety, Depression, and Stress. The total score ranges from 0 to 63. Higher scores indicate more significant distress.

The COPE [23] is a self-report questionnaire comprising 60 items rated on a 4-point Likert scale ranging from 1 to 4. It assesses different coping strategies: avoidance, Transcendent orientation, Positive attitude, Social support, and Problem orientation. Higher scores indicate greater and more significant use of the coping strategy.

The PGWBI [24] is a self-report questionnaire comprising 22 items rated on a six-point Likert scale ranging from 0 to 5. It assesses six dimensions of psychological well-being: Anxiety, Depression, Positive Well-Being, Self-Control, General Health, and Vitality. The Total Score ranges from 0 to 110. Higher scores indicate greater well-being.

Concerning DASS-21 and COPE scores, data obtained from the caregivers of subjects with PWS were compared with those recorded in the corresponding Italian normative sample of the general population (22–23). The Italian normative sample of DASS-21 was composed of 417 individuals (42.9% males), mean age ± SD: 36.39 ± 13.71, while the normative sample of the COPE was composed of 457 individuals (50% males), mean age ± SD: 49.2 ± 16.2.

### Statistical analysis

Descriptive statistics of the sample were conducted to assess frequencies and percentages for categorical variables and means and standard deviations for continuous variables. The normal distribution of the variables was evaluated by skewness and kurtosis indices. The normal distribution was guaranteed if skewness and kurtosis indices were within the acceptable range between − 2 and + 2 [25]. To assess the psychological conditions of caregivers of adult subjects with PWS, comparisons between the sample of caregivers and the control/normative samples in data of DASS-21, and COPE were performed. To assess the psychological conditions of caregivers of adult individuals with PWS, comparisons between the caregivers’ sample and the control/normative samples in DASS-21 and COPE data were performed using independent sample t-tests. The same statistic was used to compare data of adult subjects with PWS and their caregivers in PGWBI. Correlations between PGWBI, DASS-21, and COPE in the caregivers were also explored using Pearson’s correlation coefficient. Critical alpha was set at 0.05.

## Results

### Psychological conditions of caregivers of adult individuals with PWS

Data from the caregivers’ sample were compared with those obtained from the Italian validation studies of DASS-21 and COPE. Comparisons showed that the Depression (*p* < 0.001), Stress (*p* = 0.050), and Total score (*p* = 0.009) of DASS 21 were higher in the sample of caregivers than in the corresponding Italian normative sample of the general population (Table 1). No correlations were found between the Total score of DASS-21 in caregivers and the number of residents in the home (*p* = 0.739). Comparisons between caregivers and the Italian normative sample of COPE did not show significant differences (Table 2).

**Table 1:** Means, standard deviations, and t-tests of DASS-21

	Caregivers (no. 30, 19 females, 11 males)		Italian normative sample (Bottesi et al., 2015) (no. 417, 57,1% females, 42,9% males)		t	*p*
	M	SD	M	SD		
Depression	5.63	4.60	3.50	3.20	3.405	< 0.001*
Anxiety	3.07	3.68	2.40	2.60	1.320	0.187
Stress	7.83	4.68	6.40	3.80	1.958	0.050*
Total score	16.53	11.81	12.30	8.30	2.610	0.009*

*p ≤ 0.05

**Table 2:** Means, standard deviations, and t-tests of COPE

	Caregivers (no. 30, 19 females, 11 males)		Italian normative sample (Sica et al., 2008) (no. 457, 50% males)		t	*P*
	M	SD	M	SD		
Avoidance	22.1	4.30	23.5	5.1	1.469	0.142
Trascendent orientation	22.5	3.15	22.7	5.6	0.193	0.846
Positive attitude	30.1	6.29	30.9	6.0	0.705	0.480
Social support	26.0	7.06	27.7	8.4	1.129	0.259
Problem orientation	30.6	6.51	32.0	6.7	1.218	0.223

*p ≤ 0.05

### Comparison between the psychological well-being of subjects with PWS and their caregivers and correlations between coping strategies and psychological well-being in caregivers

The levels of psychological well-being of caregivers were compared with the psychological well-being of the individuals with PWS. Results showed that the PGWBI scores of caregivers were significantly lower than the PGWBI scores of the subjects with PWS in the total score (*p* = 0.006) and the different subscales. In particular, lower scores in Positive Well-being (*p* < 0.001), General Health (*p* = 0.006), and Vitality (*p* = 0.004), and higher scores in Depression (*p* = 0.005) were detected in caregivers than in the individuals with PWS (Table 3).

**Table 3:** Means, standard deviations, and t-tests of PGWBI

	Caregivers (no. 30, 19 females, 11 males)		Individuals with PWS (no. 30, 19 females, 11 males)		t	*P*
	M	SD	M	SD		
Anxiety	16.3	3.96	19.1	6.74	1.99	0.053
Depression	10.1	1.17	12.4	4.01	3.01	0.005*
Positive Well-being	10.8	3.78	15.0	4.44	3.94	< 0.001*
Self-Control	11.2	2.81	10.4	3.75	-1.01	0.315
General Health	10.2	3.06	12.5	3.14	2.88	0.006*
Vitality	12.6	4.02	15.9	4.49	2.97	0.004*
Total Score	71.2	16.06	85.2	21.85	2.83	0.006*

*p ≤ 0.05

The levels of well-being of caregivers were not associated with the psychiatric treatment of subjects with PWS (*p* = 0.539).

Regarding the psychological well-being of subjects with PWS, we explored the hypothesis suggested by the literature [26] that GH treatment could impact on well-being. An independent sample t-test comparing the total score of PGWBI of individuals with PWS undergoing GH therapy with those who were not treated with GH therapy was conducted. Results showed no significant differences (t = 1.07; *p* = 0.293) between the two subgroups. However, in our experience (data not yet published) we found that subjects with PWS scored higher than their caregivers on positive well-being, general health, and vitality.

To explore the psychological well-being of caregivers in-depth, we assessed the same hypothesis by comparing the means of the total score of PGWBI of caregivers of those subjects with PWS who were treated with GH therapy and of those who were not. Results were not significant (t = 0.18; *p* = 0.856).

We also explored the hypothesis that coping strategies could be associated with the psychological well-being of caregivers. Correlations between COPE and PGWBI revealed that the Avoidance subscale of COPE had a negative significant correlation with the total score of PGWBI (*p* = 0.003), suggesting that the higher the level of Avoidance, the lower the level of psychological well-being. Correlations are depicted in Table 4.

**Table 4:** Correlations between the subscales of COPE and the total score of PGWBI

	Avoidance		Trascendent orientation	Positive attitude	Social support	Problem orientation
Avoidance		-				
Trascendent orientation		-0.069 *p* = 0.718	-			
Positive attitude		0.025 *p* = 0.894	0.161 *p* = 0.395	-		
Social support		-0.118 *p* = 0.536	-0.118 *p* = 0.536	0.149 *p* = 0.431	-	
Problem orientation		0.156 *p* = 0.409	0.090 *p* = 0.638	0.712* *p* < 0.001	0.417* *p* = 0.022	-
PGWBI Total score		-0.520* *p* = 0.003	-0.166 *p* = 0.380	-0.213 *p* = 0.259	-0.013 *p* = 0.944	-0.301 *p* = 0.106

*p ≤ 0.05

## Discussion

The present study was aimed at assessing the psychological conditions of caregivers of adult individuals with PWS, focusing on psychological distress and coping and making comparisons between the levels of psychological well-being in caregivers and their relatives with PWS. Our results highlighted several critical insights into the profound emotional and psychological challenges faced by caregivers of subjects with PWS. In particular, the results of the study revealed that caregivers experienced high levels of anxiety and depression in comparison with the corresponding Italian normative samples (of the general population). These findings seem to confirm and extend to the adult population what previous literature underlined about the negative impact of caregiving children with PWS on caregivers’ well-being and quality of life [26, 27]. In particular, when compared to other families of children with a disability, caregivers of children with PWS reported higher levels of stress and mood disturbances, more difficulties coping with their child’s symptoms, higher levels of family conflict as well as lower quality of life [8]. This finding may be attributable to the continuous challenges and difficulties of caring for a relative with a lifelong neurodevelopmental condition [28] such as the acceptance of the child’s diagnosis and its implications, the need to provide constant supervision and support, but also the complexity of this particular syndrome in which hyperphagia is considered the most relevant factor associated with the caregiving burden [29]. Meanwhile, the financial strain of managing PWS, with its associated medical and therapeutic costs [30], may contribute to the psychological distress experienced by caregivers.

Ragusa and colleagues [19] investigated the impact of PWS on daily life and needs, and explored the resources of individuals with PWS and their caregivers, using the narrative medicine approach. The research involved twenty-one children and adolescents, thirty-four adult subjects with PWS, and one hundred and thirty-three caregivers. Most caregivers reported fatigue and required assistance to better manage the food-seeking behaviors of their children which have been considered the most challenging aspects of PWS to manage in the domestic context. Relationships external to the family were reported to be challenging to preserve, imposing a radical change in social life. A consistent number of caregivers declared that they had to change their jobs because of their children with PWS. Regarding future perspectives, PWS family caregivers reported a great concern about the future of their sons and daughters with PWS.

Furthermore, our comparison between caregivers and individuals with PWS in PGWBI provided an interesting additional confirmation of the poor psychological conditions of caregivers in PWS, with caregivers showing lower scores in Positive Well-being, General Health, and Vitality subscale and Total of PGWBI than their relatives with PWS. Subjects with PWS reported higher vitality and well-being than their caregivers. This surprising result can be explained by their living context. Individuals with PWS usually live with their families in a controlled environment and follow periodical medical visits for their entire lives, and all their familial, medical, and social resources may contribute to making them feel protected and prevent them from living significant experiences on their own, especially the negative ones.

According to our results, the caregivers’ psychological well-being did not change significantly based on the rec-GH therapy of subjects with PWS. Similarly, there were no differences between the levels of psychological well-being in patients who were treated with GH and those who were not, in contrast with the limited available literature [26]. A possible explanation for this inconsistent result could be related to the different methodological designs adopted for the different studies. The current study used a cross-sectional design to describe the present status of the sample. In contrast, the above-mentioned study of Bertella and colleagues was a longitudinal design, aimed to assess the effects of GH therapy over time and so, more suited to detect changes that might occur over a period of time.

The caregivers’ psychological well-being was associated with the use of an avoidant coping strategy, resulting in a significant negative correlation between PGWBI and the Avoidance subscale of COPE in caregivers of individuals with PWS. This result is consistent with previous work [31] elucidating that avoidance strategy was associated with poor psychological outcomes, such as low psychological well-being and high depression in caregivers of children with chronic diseases.

Our results also showed a positive and significant correlation between positive attitude and social support with problem orientation on the COPE. This result seems to confirm that people can use active coping strategies to deal with problems, such as problem-focused coping strategies based on personal characteristics including being more active than others to cope with problems by doing something active to alleviate stressful circumstances or regulate the emotional consequences of stressful events such as receiving social support or having positive attitudes to the problems [32].

In conclusion, this study highlighted the psychological conditions of caregivers of adult subjects with PWS, contributing to our understanding of the broader impact of the syndrome on the quality of life of individuals and their families.

This study is not free of limitations, including a relatively small number of participants due to the rare nature of the diseases and the lack of comparisons with other clinical populations. In addition, the study did not consider possible gender differences in coping strategies [33]given the imbalanced representation of the two genders in our study population.

Despite the above limitations, this is one of the few studies exploring the caregiver burden in adult individuals with PWS, in contrast to the vast majority of studies involving caregivers of children populations with PWS. In addition, the study holds substantial clinical relevance, highlighting the significant burden placed on caregivers. Healthcare providers and researchers should collaborate to develop and implement tailored interventions that address the specific challenges of caring for PWS individuals, prioritizing caregiver well-being as a fundamental component of PWS management. Integrating caregiver support into the healthcare system is crucial. Regular mental health screenings for caregivers should be conducted to identify those at risk of severe psychological distress. Providing resources and referrals to mental health professionals can facilitate timely intervention. Furthermore, multidisciplinary care teams should be ensured to support the caregivers and guarantee a comprehensive approach to the management of PWS.

Future research examining larger sample sizes, groups of comparison, and the impact of additional variables such as gender is required. Understanding the interplay between caregiver well-being and patient outcomes in PWS can further be investigated to provide insights into tailored intervention strategies.

## Data Availability

Raw data will be uploaded on www.zenodo.org immediately after the manuscript is accepted and will be available upon a reasonable request to the authors A.G.U. and A.S.
